# Tether fracture in leadless pacemaker during repeated recapture

**DOI:** 10.1016/j.hrcr.2022.09.012

**Published:** 2022-09-23

**Authors:** Takahiro Kusume, Yasuyuki Takada, Muryo Terasawa, Ken Takarada, Yoshinao Yazaki, Kazuhiro Satomi

**Affiliations:** Department of Cardiology, Tokyo Medical University Hospital, Tokyo, Japan

**Keywords:** Leadless pacemaker, Micra, Tether, Tether fracture, Recapture, Snare catheter, Complete atrioventricular block

**Key Teaching Points**

• The Micra transcatheter pacemaker system (Micra; Medtronic, Minneapolis, MN) is an alternative to traditional transvenous pacemakers, with high safety and efficacy. We reported a rare complication of tether fracture during Micra implantation after multiple attempts at deployment.
• Multiple repeated deployments may result in the tether fracture, following the device entrapment in the right ventricle (RV) endocardium.
• The implantation of the Micra at the basal RV endocardium has the potential risk of entrapment of the complex RV structure.

## Introduction

The Micra transcatheter pacemaker system (Micra; Medtronic, Minneapolis, MN) is a leadless pacemaker recently available in several countries and an alternative to traditional transvenous pacemakers, with high safety and efficacy.^1^ Repositioning of the Micra is required to achieve a lower pacing threshold for the long-term longevity of the battery at the device implantation. A tether, connecting the delivery catheter to the Micra, allows the Micra repositioning. The tether is strong enough to retract the Micra by a pulling-back maneuver. To the best of our knowledge, only 1 case of tether failure has been reported in the past.^2^

We report a case of tether fracture during Micra implantation after multiple attempts at deployment.

## Case report

We report a 50-year-old woman with symptomatic 2:1 atrioventricular block and acute congestive heart failure. Her comorbidities were diabetic nephropathy on maintenance hemodialysis, panic disorder, and attention deficit hyperactivity disorder.

During the management of heart failure, a complete atrioventricular block developed with a reduced heart rate of 30 beats/min on the fourth hospital day. On the eighth hospital day, the patient developed torsade de pointes owing to bradycardia-induced QT prolongation (QTc 593 ms), followed by ventricular fibrillation. Cardiopulmonary resuscitation was performed, and she was successfully resuscitated by defibrillation. The echocardiography showed normal left ventricular (LV) systolic function with LV ejection fraction 70%; LV diastolic and systolic diameter, respectively, 50 mm and 28 mm; and no valvular diseases.

Under the support of a temporary pacemaker in the right ventricle (RV), leadless pacemaker implantation was performed by the patient’s strong preference in place of the transvenous pacemaker. The delivery catheter system was advanced to the inferior vena cava through the right femoral vein.

**Figure 1:**
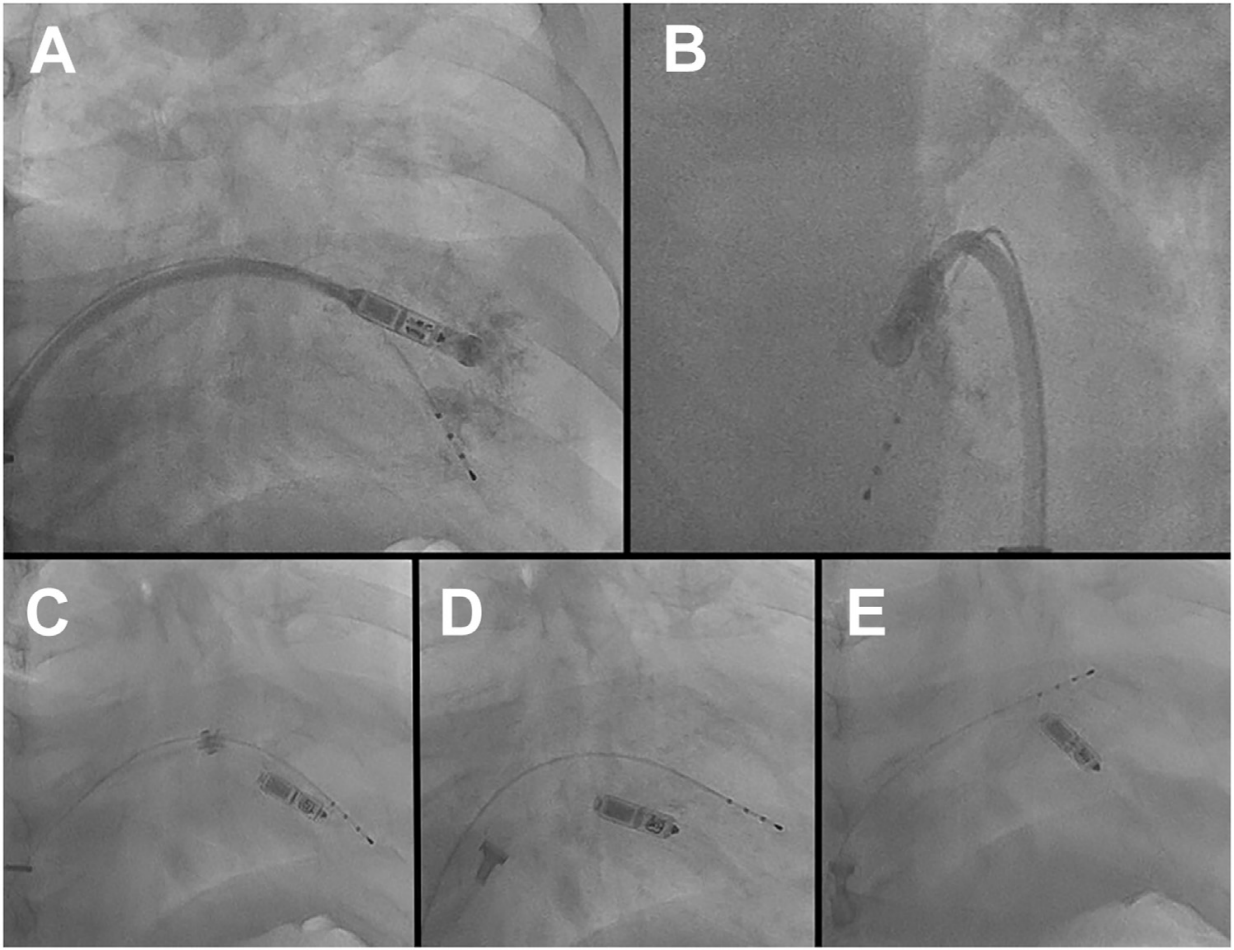
**A,B:** Right ventrile angiography demonstrated the location of the Micra pacemaker (Medtronic, Minneapolis, MN) at the initial attempt of delivery. **A:** Right anterior oblique (RAO) 30°. **B:** Left anterior oblique 60°. **C:** The position of the Micra before the tether fracture close to the tricuspid valve. **D:** The Micra cannot be stored in the recapture cone even by hard pulling. **E:** The tether has finally broken during continuous pulling of the tether. (C–E: RAO 30°.)

The Micra was deployed in the septal side of the apex at the first attempt (Figure 1A and 1B). The threshold value was high (>2.0 V / 0.4 ms) and we changed the implantation site to the lower or middle septum and basal septum near the tricuspid annulus (Figure 1C). The threshold was still high at the seventh attempt at deployment.

After the seventh attempt, the retraction of the Micra was no longer possible, even keeping the head of the Micra coaxial with the recapture cone.

Then we tried to retrieve the Micra by moving the position of the tip of the sheath. However, the head of the Micra could not be stored in the recapture cone (Figure 1D).

We thought that the Micra was entangled in the complex structure in the RV basal endocardium and could not be pulled out with normal force. During continuous pulling of the tether with strong power, the tether finally was broken (Figure 1E).

**Figure 2:**
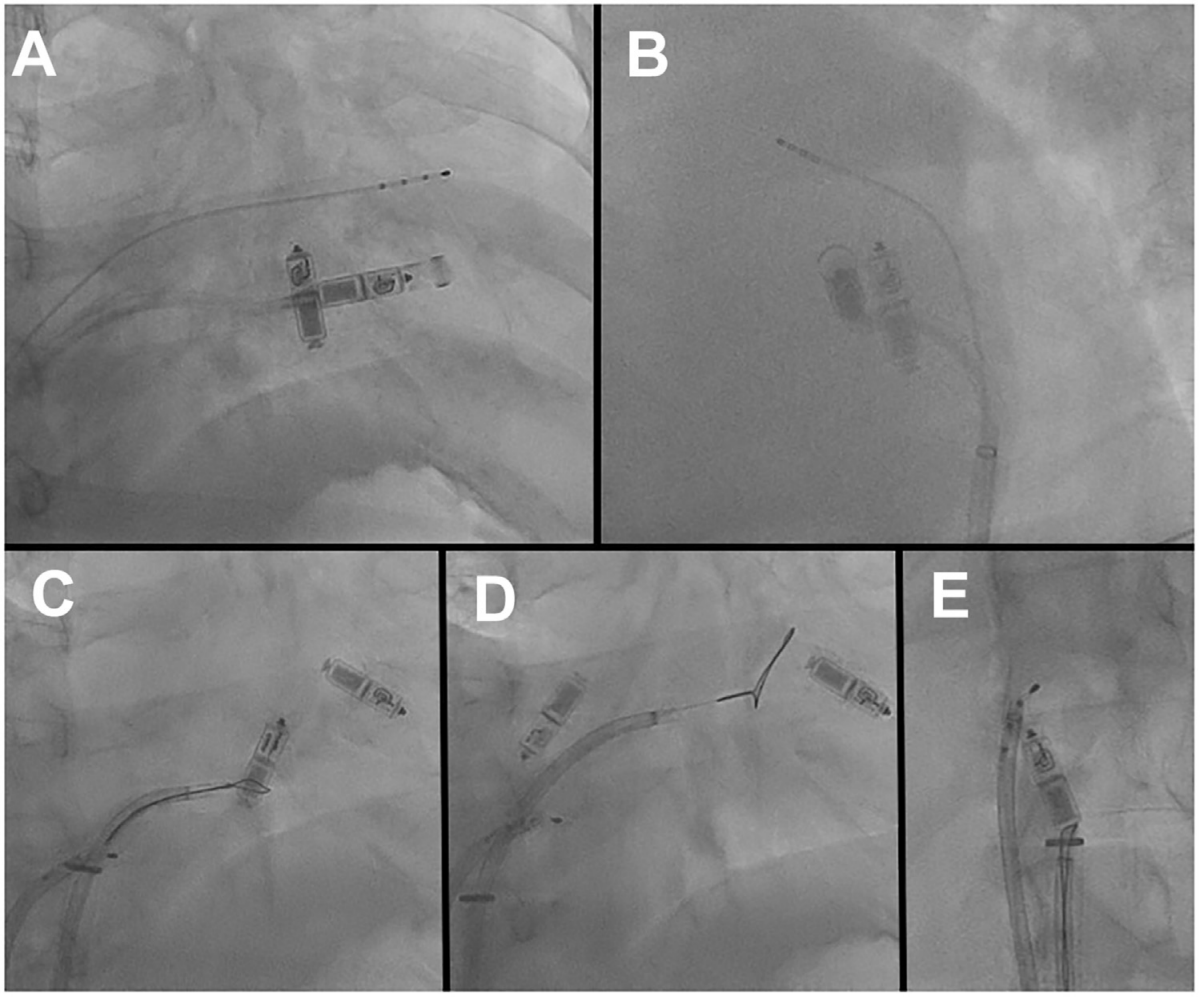
**A,B:** The deployment of the second Micra pacemaker (Medtronic, Minneapolis, MN) at high right ventricle septum (A: right anterior oblique [RAO] 30°; B: left anterior oblique 60°). **C:** The goose-neck snare caught the tail of the Micra supported by the deflectable sheath. **D:** Strong traction finally dislodged the Micra from the myocardium. It moved into the right atrium on the spur of movement. **E:** The Micra was caught with a snare in the superior vena cava and was successfully retrieved. (C–E: RAO 30°.)

We used another Micra system and finally implanted the Micra in the high septum after 6 attempts with a better pacing threshold (0.38 V / 0.4 ms) (Figure 2A and 2B). After that, we successfully retrieved the first locked Micra using a deflectable sheath (Agilis; Abbott, Plymouth, MN) and 25 mm snare catheter (Amplatz Goose Neck Snare; Medtronic) (Figure 2C–2E).

**Figure 3:**
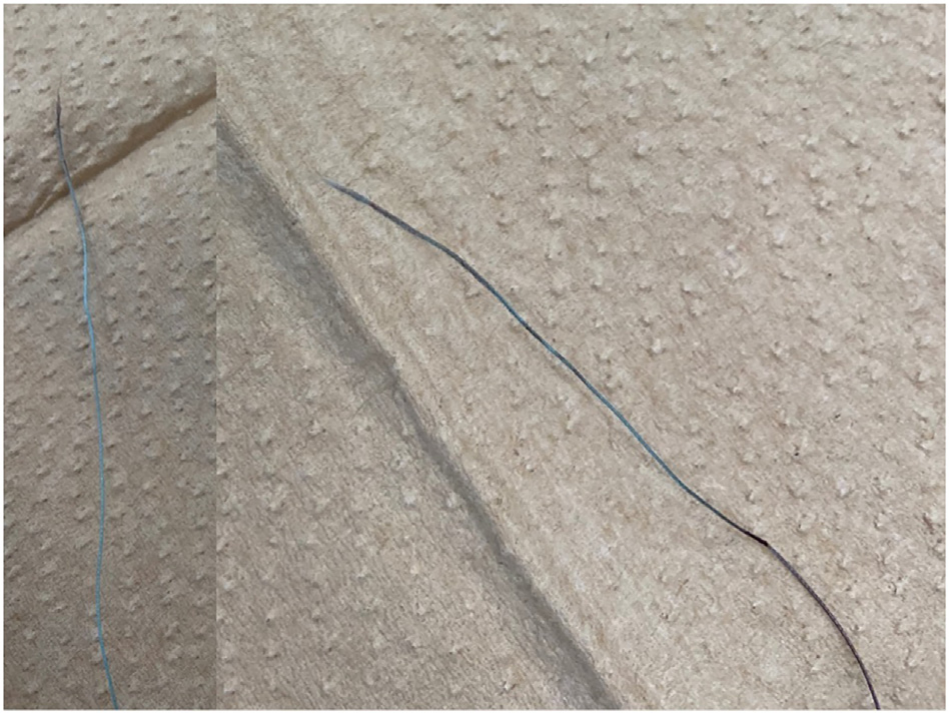
The broken tether. No abrasion was observed.

Finally, we observed the broken tether had no abrasion (Figure 3). After Micra implantation, the patient became ventricular pacing dependent. An echocardiogram did not show the apparent tricuspid regurgitation.

During 13 months of follow-up after implantation, the pacing threshold was still acceptable (0.25 V / 0.24 ms) and the electrocardiogram showed normal QTc of 420 ms. Also, there were no episodes of syncope or high ventricular rate episodes in the clinical course.

## Discussion

We reported a rare complication of tether fracture of a Micra pacemaker. More than 100,000 Micras have been implanted since the first Micra leadless pacemaker was launched. Only 1 case report was published concerning tether fracture.^2^

The tether is made of polyether and coated with polytetrafluoroethylene. It can withstand very high tension and does not fail during normal retrieval procedures. From the data of the manufacturer, the strength of the tether is expected to be a weight of 2 kg.

In previous reports, more than 6 redeployments frequently lead to pericardial effusions in high-risk patients.^3^ In our case, the deployment of the Micra was difficult, and we attempted 7 deployments before the fracture of the tether. After the seventh deployment at the basal septum, the Micra was never retracted to the recapture cone. Repeated recapture may increase the risk of retraction failure.

The basal septum of the RV has a complicated anatomical structure with the tendon chordae and papillary muscle of the tricuspid valve. This complicated anatomical structure was related to the catheter entrapment in the tricuspid valve, as already reported.^4^ We speculated that the Micra became entangled and fixed in a complex RV basal endocardium during the deployment process. As a result, the tether broke when pulled with a strong force beyond the durability of the tether. Fortunately, the successful extraction of the fixed Micra was possible without the destruction of the tricuspid valve.

A previous report described that the tether was weakened by abrasion owing to repeated retraction if the angle between the device and the recapture cone was too steep during recapture.^2^ The tether had no abrasion in this case; therefore we did not consider this phenomenon as the reason for tether fracture. Another explanation is the excessive force over the durability of the tether, which was weakened by repeated deployment. Fixed entrapment of the Micra in the complex RV endocardium structure and the tether weakened by repeated redeployment may be the underlying cause of the fracture of the tether in this case.

## Conclusion

We report a case of tether fracture during Micra implantation. The implantation of the Micra at the basal RV endocardium has the potential risk of entrapment of the complex RV structure. In addition, the repetitive recapture probably caused a risk for tether fracture.
